# Circulating cardiomyocyte-derived extracellular vesicles reflect cardiac injury during systemic inflammatory response syndrome in mice

**DOI:** 10.1007/s00018-021-04125-w

**Published:** 2022-01-20

**Authors:** Hargita Hegyesi, Éva Pallinger, Szabina Mecsei, Balázs Hornyák, Csenger Kovácsházi, Gábor B. Brenner, Zoltán Giricz, Krisztina Pálóczi, Ágnes Kittel, József Tóvári, Lilla Turiak, Delaram Khamari, Péter Ferdinandy, Edit I. Buzás

**Affiliations:** 1 Department of Genetics, Cell- and Immunobiology, Semmelweis University, Budapest, Hungary; 2 Department of Pharmacology and Pharmacotherapy, Semmelweis University, Budapest, Hungary; 3 Institute of Experimental Medicine, Eötvös Loránd Research Network, Budapest, Hungary; 4 Department of Experimental Pharmacology, National Institute of Oncology, Budapest, Hungary; 5 MS Proteomics Research Group, Research Centre for Natural Sciences, Eötvös Loránd Research Network, Budapest, Hungary; 6 ELKH-SE Immune-Proteogenomics Extracellular Vesicle Research Group, Budapest, Hungary; 7 Pharmahungary Group, Szeged, 6722 Hungary; 8 Hungarian Centre of Excellence for Molecular Medicine (HCEMM), Semmelweis University Extracellular Vesicle Research Group, Budapest, Hungary

**Keywords:** Extracellular vesicles, Cardiomyocyte, Cardiomyopathy, Inducible transgenic mice, SIRS

## Abstract

The release of extracellular vesicles (EVs) is increased under cellular stress and cardiomyocyte damaging conditions. However, whether the cardiomyocyte-derived EVs eventually reach the systemic circulation and whether their number in the bloodstream reflects cardiac injury, remains unknown. Wild type C57B/6 and conditional transgenic mice expressing green fluorescent protein (GFP) by cardiomyocytes were studied in lipopolysaccharide (LPS)-induced systemic inflammatory response syndrome (SIRS). EVs were separated both from platelet-free plasma and from the conditioned medium of isolated cardiomyocytes of the left ventricular wall. Size distribution and concentration of the released particles were determined by Nanoparticle Tracking Analysis. The presence of GFP + cardiomyocyte-derived circulating EVs was monitored by flow cytometry and cardiac function was assessed by echocardiography. In LPS-treated mice, systemic inflammation and the consequent cardiomyopathy were verified by elevated plasma levels of TNFα, GDF-15, and cardiac troponin I, and by a decrease in the ejection fraction. Furthermore, we demonstrated elevated levels of circulating small- and medium-sized EVs in the LPS-injected mice. Importantly, we detected GFP^+^ cardiomyocyte-derived EVs in the circulation of control mice, and the number of these circulating GFP^+^ vesicles increased significantly upon intraperitoneal LPS administration (*P* = 0.029). The cardiomyocyte-derived GFP^+^ EVs were also positive for intravesicular troponin I (cTnI) and muscle-associated glycogen phosphorylase (PYGM). This is the first direct demonstration that cardiomyocyte-derived EVs are present in the circulation and that the increased number of cardiac-derived EVs in the blood reflects cardiac injury in LPS-induced systemic inflammation (SIRS).

**Supplementary Information:**

The online version contains supplementary material available at 10.1007/s00018-021-04125-w.

## Introduction

Cardiovascular diseases represent the number one cause of death worldwide. Despite major research efforts focusing on cardiovascular diseases, there is still a demand for early and sensitive diagnostic markers for myocardial injury. Extracellular vesicles (EVs) are subcellular, phospholipid-bilayer enclosed particles present both in tissues and biological fluids [1]. Recently, outstanding interest has been attracted by EVs as a potential next-generation biomarker. EVs have been shown to cross biological barriers and serve as promising complex diagnostic and prognostic tools [2]. Secreted by various types of cells, EVs carry important biomolecules originating from their parent cells. The guidelines of the International Society for Extracellular Vesicles (MISEV2018) recommended referring to EVs either by their physical or molecular characteristics given that in most studies, the precise biogenesis route of EVs is not investigated and confirmed [3].

It was tempting to speculate that cardiovascular diseases could be monitored based on circulating cardiomyocyte-derived EVs. However, direct evidence for the presence of heart-derived EVs in the systemic circulation has been lacking. Here we applied a conditional double transgenic mouse model in which cardiomyocytes show green fluorescence, while all other cells of the body express the red tdTomato [4]. The mTmG reporter transgene is driven by an beta-actin promoter from the Gt(ROSA)26Sor genomic locus. In the absence of Cre recombinase, mTmG mice constitutively express tdTomato [5]. Exposure to Cre recombinase occurs upon crossing the mTmG mice with MerCreMer mice, which express tamoxifen-inducible Cre driven by the alpha-myosin heavy chain promoter [6]. Following tamoxifen induction, the tdTomato expression cassette is excised, and the rearranged mTmG transgene converts to the expression of the enhanced green fluorescent protein (eGFP). Cardiomyocyte-targeted MerCreMer transgenic mice have been used to control gene expression in the heart [7].

To monitor cardiomyopathy, we used an LPS-induced systemic inflammatory response syndrome (SIRS) model [8, 9]. Sepsis-induced cardiomyopathy is a global but reversible cardiac dysfunction that features left ventricular dilatation and reduction of the ejection fraction. In this in vivo model, it has been reported that increased levels of pro-inflammatory cytokines and oxidative stress can induce mitochondrial damage and cardiac cell death [10]. Our data presented here provide evidence for an increased release of cardiomyocyte-derived EVs to the circulation in the LPS-induced cardiomyopathy model.

## Methods

### Mice

C57BL/6 mice were purchased from the Jackson Laboratories (Bar Harbor, ME, USA). Myh6-Cre tamoxifen-inducible transgenic mice (MerCreMer Stock: 005657) expressing Cre recombinase under the control of the Myh6 promoter and mTmG mice (Stock: 007576) were purchased from Jackson Laboratories (Bar Harbor, ME, USA). ROSAmTmG is a cell membrane-targeted, two-color fluorescent Cre-reporter allele. Prior to Cre recombination, cell membrane-localized tdTomato (mT) red fluorescence expression is widespread in all the cells. Cre recombinase expressing cardiomyocytes have cell membrane-localized EGFP (mG) fluorescence expression replacing the red fluorescence. Tamoxifen (Sigma-Aldrich) induction of Cre gene expression was carried out by intraperitoneal [75 mg/kg body weight in corn oil (Sigma-Aldrich)] injections for 5 consecutive days, while control mice received 0.9% NaCl injections. One week later, 6 mg/kg LPS (O111:E4 Sigma-Aldrich) or 0.9% NaCl (as a control) were administered intraperitoneally. Genotyping was performed upon tail clipping with DNA polymerase chain reaction (PCR with REDTaq^®^ ReadyMix, Sigma-Aldrich) as recommended by the supplier. All animal studies were conducted according to the 1998 XXVIII Hungarian law about animal protection and welfare. The ethics approval number is PE/EA/1426-7/2018. Mice have been kept in individually ventilated cages, with 12 h light: 12 h dark cycles. Mice were used at the age of 12–14 weeks of age. The experiments were conducted with the approval of the Semmelweis University Animal Care and Use Committee.

### Primary adult mouse cardiomyocyte culture

Mice were euthanized by carbon dioxide inhalation. The chest was opened, and the heart was rapidly removed and placed in an ice-cold HBBS solution (Thermo Scientific, USA). Cell isolation was performed based on a previously published protocol [11] with minor modification. The tissue was digested with collagenase II and protease XIV (both from Sigma-Aldrich), according to the manufacturer’s protocol. Separated cells were filtered through a 70 µm mesh filter (Fisher Scientific) and cardiomyocytes were allowed to settle by gravity for 15 min in a 10 mL tube. After 15 min, the upper 9 mL was carefully discarded, and the bottom single-cell suspension (1 mL) was plated and incubated in a complete cardiomyocyte medium in a T25 flask. The complete cardiomyocyte medium was DMEM (from the Pierce Primary Cell Isolation kit, Thermo Scientific) supplemented with 10% fetal bovine serum (Biosera, Biotech, Prague, Czech Republic), and 1% (v/v) streptomycin/penicillin (Sigma-Aldrich). The cells were incubated in a T25 flask (Eppendorf)/heart for 1 h. The non-adherent cells were then transferred to another T25 flask (Eppendorf) and were cultured for 24 h. Necrotic cell death was assessed by microscopic observation of Trypan blue exclusion and was ∼ 80%. This second T25 flask was coated with 1 µg/mL fibronectin (Sigma-Aldrich).

### Isolation of EVs from blood plasma and conditioned media of primary adult cardiomyocyte cultures

Mice were sacrificed with CO_2_ and ACD (acid citrate dextrose) anticoagulated blood was collected from the inferior vena cava with a needle and a syringe. Plasma samples were centrifuged at 800×*g* for 5 min to sediment the cells. The supernatant was removed, and platelet-poor plasma (PPP) was prepared by centrifugation at 2500×*g* for 15 min, at room temperature. Platelet free plasma (PFP) was then obtained from PPP samples by centrifugation at 2500×*g* for 20 min at 16 °C (Z216 MK Microlite centrifuge, fixed angle 200.88 rotor, Hermle Labortechnik, Germany). Medium-sized EV-enriched preparations (we further refer to them as mEVs) were pelleted by centrifugation at 12,500×*g* for 20 min [3]. The mEVs were washed with PBS and resuspended in 100 µL PBS for further analysis. The supernatant was next filtered by gravity through a 0.2 µm filter and pelleted at 100,000×*g* for 70 min at 4 °C using an MLA-55 rotor in an Optima Max XP ultracentrifuge (Beckman-Coulter). The small EV (sEV)-enriched pellet (further referred to as sEVs) was washed once with PBS and re-suspended in 50 μL PBS. The protein content of the mEV- and sEV-enriched preparations was determined by the Micro-BCA assay (Pierce).

Mouse cardiomyocytes were cultured in complete DMEM supplemented with 2.5% EV-free fetal calf serum (Biosera). After 24 h, a 5 mL conditioned medium was centrifuged at 600×*g* for 10 min to remove cells. This was followed by gravity-driven filtration through a 5 µm filter (Millex, Millipore) and centrifugation by 2000×*g* for 5 min at RT (the pellet contained large EVs lEVs [12]. Next, the supernatant was subjected to filtration through a 0.8 µm filter (Nalgene Syringe Filter, Thermo Scientific), and mEVs were pelleted at 12,500×*g* for 30 min (the resulting pellet was enriched in mEVs [13]. The separation of small EVs was conducted the same way as described above (100,000×*g* for 70 min at 4 °C using an MLA-55 rotor).

#### Purification of PFP-derived EVs by size exclusion chromatography (SEC)

The starting volume of platelet-free plasma used for each isolation was 0.2 mL. Before loading the samples onto the column, they were diluted five times. SEC columns (qEV single (IZON), separation size: 70 nm Ser: Y1000931) were used according to the instructions of the manufacturer. As elution buffer, EV buffer (0.2 μm filtered 0.9% NaCl containing 10 mM HEPES, pH 7.4) was used after performing the recommended column equilibration. We collected 200 µL fractions and pooled 1–3, 4–6, 7–9 and 10–12 fractions (600 μL/each pool) and analyzed them by flow cytometry. The pooled fractions were then spun at 12,500 g for 20 min, the pellets were re-suspended in 10 μL EV buffer and were subjected to Cytoflex analysis. Only fractions 4–6 contained CD63-APC positive, Triton-sensitive events (Triton X-100, 0.1%).

### Flow cytometry

Flow cytometry was used for the characterization of circulating EVs. Samples were assessed by a FACSCalibur flow cytometer (BD, San Jose, CA, USA) and data were analyzed by CellQuestPro software (BD, San Jose, CA, USA). In experiments with MerCreMer/mTmG mice, for multicolor flow cytometry, we used a Cytoflex flow cytometer (Beckman Coulter). For mEV analysis, Megamix-Plus SSC (BioCytex Marseille, France) calibration beads and 1 μm Silica Beads Fluo-Green Green (Kisker Biotech GmbH & Co; Steinfurt, Germany) were used for the optimization of cytometer settings and also for the definition of “EV gate”. mEV samples were divided into 300 μL aliquots and were incubated with fluorochrome-conjugated antibodies for 15 min at room temperature for immunophenotyping of mEVs. Annexin V (SONY) staining was carried out in a 2.5 mM Ca^++^ containing Annexin binding buffer. Unstained samples and EV-free antibody solutions (staining controls) were used for the evaluation of fluorescence background according to MISEV2018 [3]. Aspecific binding of fluorochrome-conjugated antibodies was assessed by using isotype control immunoglobulins. Differential detergent lysis by 0.1% Triton X-100 (Sigma-Aldrich) was used to confirm the vesicular nature of events, as described by György et al. [14]. The absolute number of mEVs was determined by adding counting beads (Count Check Beads; Partec, Germany) to the mEV samples diluted in PBS. The absolute number of mEVs was calculated by the following formula:

Absolute mEV Count (mEVs/μL) = [(detected mEV events inside the EV gate—Triton X-100 resistant events)/(acquired bead number inside the bead gate) × (Count Check bead number in the tube/plasma dilution)]. Cytoflex (Becton) flow cytometry was used for the characterizations of circulating EVs isolated from double transgenic mice (MerCreMer/mTmG).

In all flow cytometry tests, EV pellets were resuspended in 100 µL buffer, and 10 µL was mixed with 290 µL of diluted antibodies. A list of the used antibodies with all details (lot number, catalog number, and dilution) is provided in Supplementary Table 1.

#### Intravesicular labelling of Troponin I

Flow cytometric detection of GFP + and TroponinI + mEVs was carried out using a Cytoflex flow cytometer (Beckman Coulter). For the detection of mEVs, anti-Troponin I ((cardiac muscle TNNI3 antibody (Biotin), abbexa)) and streptavidin-APC (Sony) staining was used. Dye‐only and unstained controls were also tested. For the determination of the vesicle concentration, count check beads were applied (Sysmex). After SEC purification, the re-isolated mEVs (12,500×*g* 20’) were resuspended in 100 μL of filtered EV buffer supplemented with saponin (10 mM HEPES containing 0.9% NaCl, 0.1% saponin pH 7.4) before the addition of either anti-troponin I-biotin conjugated antibody or the isotype control. After 20 min incubation at room temperature, the mEV suspension was diluted with 1 mL of filtered EV buffer before centrifugation at 12,500×*g* for 20 min to remove non-bound antibodies. The pellets were resuspended and incubated with streptavidin-APC in 100 μL filtered EV buffer for 20 min at room temperature. All mEV pellets were resuspended in 1.0 mL filtered PBS before a final centrifugation (12,500×*g*, 20 min). The pellets were resuspended again in 300 μL of filtered PBS before flow cytometry. To prove the vesicular nature of the detected events, lysis control with 0.1% Triton was applied for 5–10 min [14].

### Labeling of CMEVs with MitoTracker red

CMEVs were isolated from the conditioned medium and the EV pellet was resuspended in 50 µL of PBS. CMEVs were labeled with 100 nM final concentration of MitoTracker Red (ThermoFisher,) in PBS. Samples were stained for 15 min at 37 °C in the dark, followed by PBS washing and centrifugation with 12,500×*g* for 20 min followed by analysis by Cytoflex.

### Trypsin and Triton-X treatment

CMEVs were diluted to 10 μg/mL protein concentration and incubated in the presence of 20 μg/mL Trypsin (Thermo) or 0.01% Triton X-100 in PBS for 1 h at 37 °C with gentle vortexing every 15 min. Following this, EVs were immediately labeled for flow cytometry as described above.

### Transmission electron microscopy

Murine cardiomyocyte-derived mEV and sEV pellets were fixed with 4% paraformaldehyde in PBS, washed with PBS, and post-fixed in 1% OsO_4_ for 30 min as described previously [15]*.* After rinsing with distilled water, pellets were dehydrated in graded ethanol, including block staining with 1% uranyl-acetate in 50% ethanol for 30 min, and were embedded in Tab 812. Overnight polymerization of the samples was carried out at 60 °C, and it was followed by ultrathin sectioning and analysis using a Hitachi 7100 transmission electron microscope (Hitachi Ltd).

### Fluorescent microscopy

For imaging cells from animals upon necropsy and for ex vivo imaging of cryostat sectioned mouse organs (~ 3 µm thickness), we used fluorescent microscopy (Eclipse E600, Nikon) and a CCD camera (Nikon). For imaging of tdTomato fluorescence with 554 nm excitation and 581 nm emission maxima, we used red fluorescent protein (RFP) filter sets. For green fluorescent protein (GFP) imaging with 484 nm excitation and 510 nm emission maxima, we used 475/40 excitation and 530/50 bandpass emission filters.

### Hematoxylin and eosin (H&E) staining

The mice were bled out by the puncture of their inferior vena cava. The hearts were excised, and the atria were removed under sterile ice-cold HBBS. Then the left and right ventricles were separated by a sterile scalpel blade. Finally, 2 mm thick pieces of the left ventricle wall were dissected and fixed immediately in 4% paraformaldehyde for 24 h and embedded in paraffin. Sections of 4 µm thickness were stained with H&E. The number of inflammatory cells was counted in 6 randomly chosen histological fields (6 control and 6 LPs mice × 2 sections × mean of 6 fields). Image analysis was done by ImageJ software (https://imagej.nih.gov/ij/).

### Enzyme-linked immunosorbent assay

The levels of tumor necrosis factor-alpha (TNFα) (Sigma), growth differentiation factor 15 [GDF-15 (Abcam)], and cardiac troponin I (cTnI) (myBiosource) in platelet free plasma (PFP) of mice were quantified by commercial ELISA kits following the manufacturer’s instructions. The plates were read by a FluoroSkan Plate Reader, and the absorbance was measured at 450 nm.

### Capillary electrophoretic-based immunoassay (WES)

mEVs were isolated with differential centrifugation then were washed with PBS and lysed in 10X diluted CelLytic M buffer (Sigma) and 1 μL Complete Protease Inhibitor Cocktail (Roche). The lysates were sonicated for 10 min, centrifuged at 14,000×*g* for 15 min, and protein concentrations of the supernatants were measured with the Micro BCA Protein Assay Kit (Thermo Fisher Scientific) 2 μg protein were applied to capillary-based Simple Western analysis WES (ProteinSimple, San Jose, CA) according to the manufacturer's instructions. SM-W004 (for analysis between 12 and 230 kDa), DM-TP01 total protein detection kit, DM-001 anti-rabbit detection kit, and PS-ST02EZ-8 EZ Standard Pack 2. The results were analyzed by the Compass for SW 4.0.1 software (ProteinSimple).

### Echocardiography in mice

Anesthesia of 10–12 weeks old male C57BL/6 mice included was induced by 5% and maintained by 2% isoflurane. Mice were scanned in a supine position on an automatic heating pad by using a micro-ultrasound imaging unit (Vevo 3100 imaging system VisualSonics) equipped with an ultrahigh-frequency MX400 transducer (30 MHz, 55 frames per second). Standard two-dimensional B- and M-mode echocardiographic measurements were performed in parasternal long- and short-axis views. Diastolic parameters were evaluated in the apical 4-chamber view by recording maximal transmitral flow velocities (pulsed-wave Doppler) and mitral annular velocities (tissue Doppler). Measured parameters included heart rate (HR), LVAWd (left ventricular anterior wall thickness in diastole), LVAWs (left ventricular anterior wall thickness in systole), LVPWd (left ventricular posterior wall thickness in diastole), LVPWs (left ventricular posterior wall thickness in systole), LVIDd (left ventricle internal diameter in diastole), LVIDs (left ventricle internal diameter in systole), peak velocity of blood inflow across the mitral valve during early diastole (E), isovolumetric relaxation time (IVRT), isovolumetric contraction time (IVCT), peak early-diastolic annular velocity (e’). Calculated parameters included volume calculations (LVEDV (left ventricular end-diastolic volume) and LVESV (left ventricular end-systolic volume) were calculated from the rotational volumes of the left ventricular trace at the diastole and systole around the long axis line of the spline), stroke volume (SV as LVEDV-LVESV), ejection fraction (EF% as (LVEDV-LVESV)/LVEDVx100), fractional shortening (FS% as (LVIDd-LVIDs)/LVIDdx100), cardiac output (CO as SVxHR/1000), left ventricle mass (LV mass using Devereux’s formula modified for rodents as 1.04 [(LVIDd + LVAWd + LVPWd)^3^ − LVIDd^3^] × 0.8 + 0.6) and the ratio of E and e’ (E/e’).

Echocardiographers and evaluators were blind to the treatment of mice. Images were analyzed by image analysis software (Vevo LAB Software; VisualSonics).

### Nanoparticle tracking analysis (NTA)

Particle number and particle size distributions were determined by using an NTA instrument (Zetaview, Particle Metrix, Germany). All samples were diluted in PBS to a final volume of 1 mL. Optimal measurement concentrations were determined (180–400 particles/frame). The manufacturer’s default software settings were selected for EV analysis. For each measurement, two cycles were performed by scanning 11 cell positions and were captured under the following settings for mEVs: Focus: autofocus; Camera sensitivity for all samples: 75.0; Shutter: 70; Cell temperature: 25 °C. After capture, the videos were analyzed by the in-build ZetaView Software 8.05.11 SP1 with specific analysis parameters: Maximum area: 1000, Minimum area 5, Minimum particle brightness: 20. Small EVs were determined at 80 sensitivities; Shutter: 100; with specific analysis parameters: Maximum area: 200, Minimum area 10, Minimum particle brightness: 30.

### Statistical analysis

GraphPad Prism version 8.0 (GraphPad Software, La Jolla California, USA) was used for statistical analysis after testing for data normality (D’Agostino-Pearson normality test). Two-sided Student’s unpaired *T*-test was used for normally distributed data, and ANOVA analysis followed by Tukey’s multiple comparisons test was carried out for multiparameter analysis. Significance was defined as *P < 0.05.

## Results

### Tamoxifen-inducible conditional transgenic mice express GFP only in cardiomyocyte cells

**Fig. 1:**
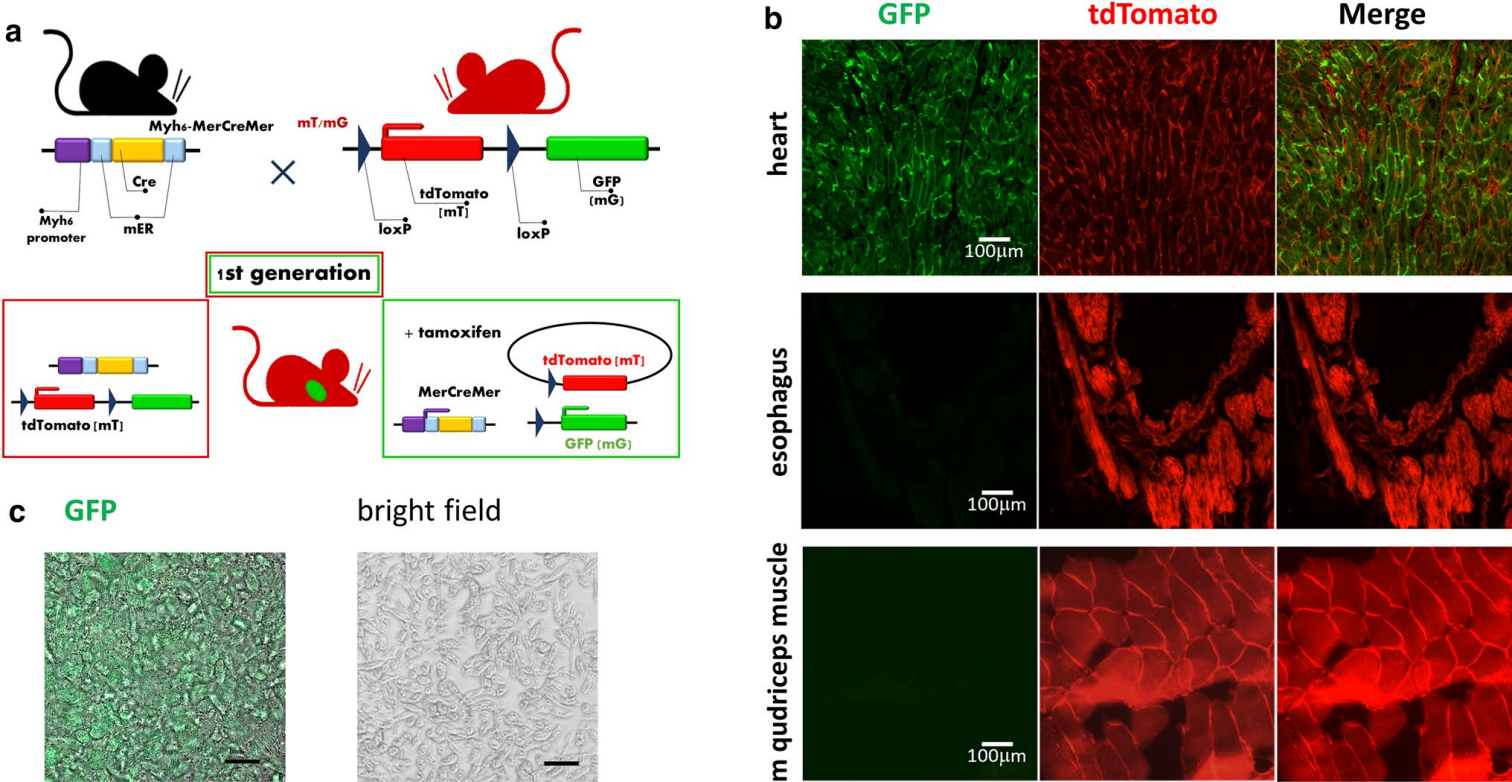
Tissue-specific and inducible Cre-mediated expression of GFP in cardiomyocytes. **a** Generation of tamoxifen-inducible double transgenic animals (MerCreMer/mTmG). Schematic illustration of MerCreMer and mTmG construct to generate bi-transgenic animals. MerCreMer mice are crossed with mTmG animals to trace the lineage of GFP-positive cells in the cardiomyocyte. **b** Ubiquitous expression of tdTomato and cardiomyocyte-specific expression of GFP demonstrated in mouse tissue from a MerCreMer x mTmG cross at necropsy. Fluorescent, F1 generation littermates were euthanized and imaged at 12 weeks of age. All sections were recorded during a single imaging session using the same exposure times. GFP expression is limited to the cardiomyocyte whereas tdTomato is brightly expressed in all the other cells. The images of different muscle tissues were adjusted equally for brightness and contrast. All other images are shown without post-acquisition processing. Scale bars, 100 μm. **c** Cardiomyocytes were isolated from adult transgenic mice. The left panel shows the isolated GFP expressing cardiomyocytes under the 20X objective, the right panel shows the bright field image of the isolated single cells. Scale bars: 30 µm

We used mTmG (B6;129-Gt(ROSA)26Sortm11(CAG-tdTomato*,-GFP*)Nat/J) indicator mice (Jackson Laboratories), engineered to constitutively express a conditional tdTomato transgene that converts to green fluorescent protein (GFP) expression following exposure to Cre recombinase. We crossed the mTmG mice with the MerCreMer (B6.FVB(129)-A1cfTg(Myh6-cre/Esr1*)1Jmk/J) transgenic mice (Jackson Laboratories). To confirm the phenotype, we performed fluorescence imaging of the heart, esophagus, and striated muscle sections from F1 offsprings of MerCreMer and mTmG crosses (Fig. 1a). MerCreMer/mTmG mice demonstrated bright GFP expression in cardiomyocytes and intense tdTomato expression in all other cells. As both GFP and tdTomato are membrane-targeted, individual cell borders were visible in the fluorescent images (Fig. 1b). Then we isolated cardiomyocytes to demonstrate cardiomyocyte-specific GFP expression using conventional fluorescent microscopic images. Viable cardiomyocytes from adult heart ventricles were isolated, two steps enzymatic digestion from a 12-week-old mouse heart was performed with a standard protocol. Figure 1c shows a representative image of a rod-shaped cardiomyocyte under a bright-field (right panel) and the green channel (left panel). Immunofluorescence images were obtained by Cell Discoverer 7 (Zeiss).

### LPS induces inflammation, EV release, and cardiac dysfunction

**Fig. 2:**
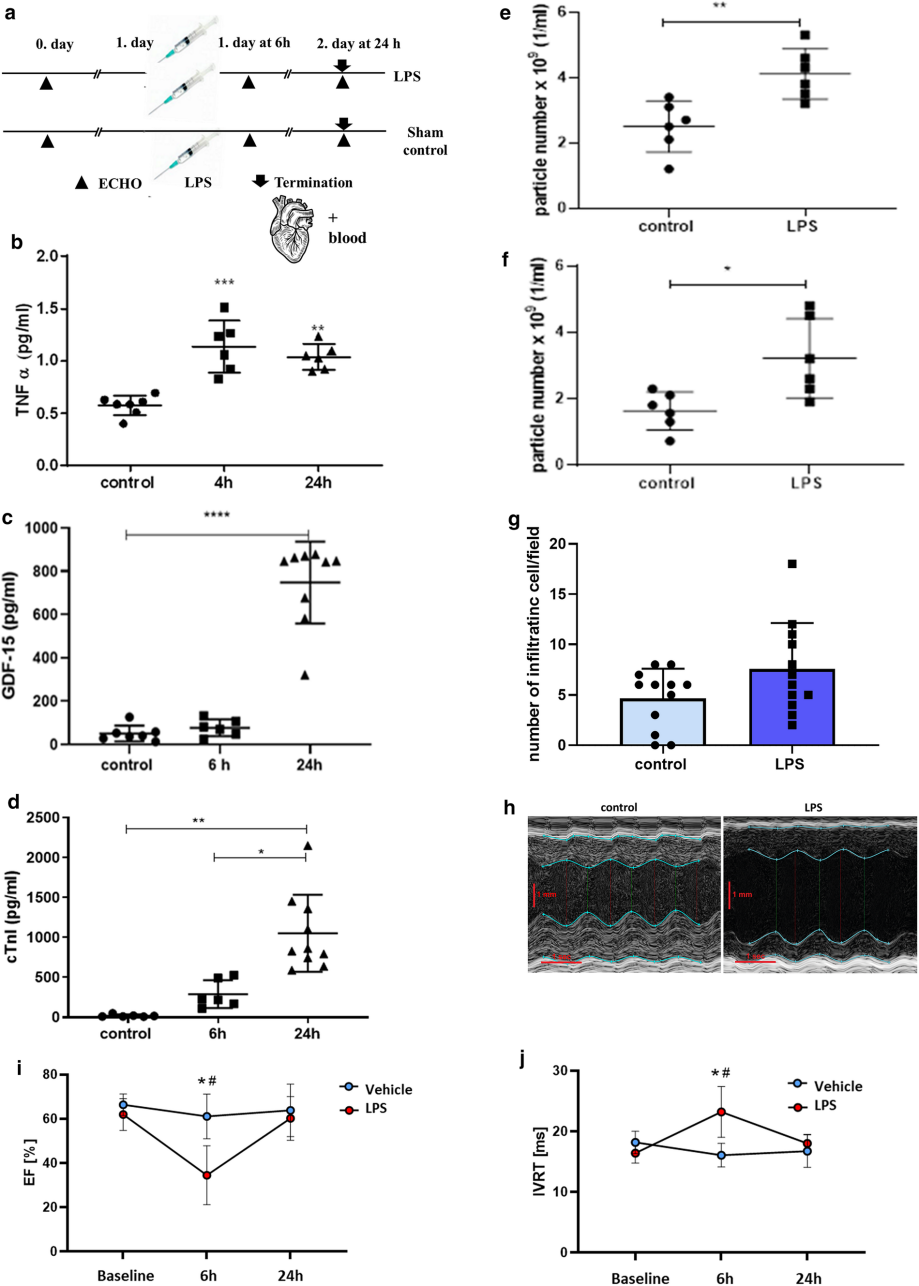
Effect of an LPS injection on inflammation, EV release, and cardiac function. **a** C57/Bl6 mice were injected intraperitoneally with 6 mg/kg of LPS/mouse. **b** TNF-α in platelet-free plasma (PFP) (*n* = 6–8). **c** GDF-15 in PFP (*n* = 6–8). ELISA results after 4 h/6 h and 24 h after LPS injection. **d** Cardiac Troponin I was measured by ELISA 6 h and 24 h after LPS injection. Data points represent values from individual mice (means ± SD are indicated). *P < 0.05; **P < 0.01; ***P < 0.005; ****P < 0.001). **e**, **f** mEVs were isolated from 200 µl of mouse PFP using differential centrifugation e) followed by ultracentrifugation to separate sEVs **f** *n* = 6. Particle concentration was measured by NTA 6 h and 24 h after the treatment. **g** The graph shows the number of infiltrating cells/fields for each condition. Quantitative results indicate the average values ± SD of *n* = 3 animals in each group. The results were analyzed by Student’s *T*-test, no significant difference was found. **h** Representative images of the echocardiography analysis of the mouse hearts 6 h after LPS injection. **i**, **j** LPS led to a significant reduction of EF (ejection fraction) and prolongation of IVRT (isovolumic relaxation time). Data were analyzed using two-way ANOVA followed by Tukey's multiple comparisons test. Data are presented as mean ± SD (*P < 0.05 vs vehicle 6 h, ^#^*P* < 0.05 vs LPS baseline)

We induced murine sepsis by intraperitoneal administration of LPS (6 mg/kg). It has been demonstrated that in this model, septic cardiomyopathy is induced (see Fig. 2a for experimental setup) [16]. To confirm that our LPS treatment induced a systemic inflammation and subsequent EV release to the circulation, we measured the levels of TNFα, GDF-15 by conventional ELISAs from platelet free plasma (PFP). We also determined the particle concentrations in isolated mEV and sEV preparations from PFP samples 24 h after LPS injection by NTA. As shown in Fig. 2b, c, following an LPS challenge, the levels of TNFα and GDF-15 were indeed elevated as compared to controls at various time points after LPS injection. Moreover, LPS treatment resulted in an increased particle number in the mEV and sEV preparations (Fig. 2e, f and Supplementary Fig. 1 a–c). These results were consistent with the LPS-challenged inflammatory response [17]. Cardiac troponin I (cTnI) is a known blood plasma marker of cardiomyocyte damage [18]. The mean plasma cTnI concentration was elevated at 6 h and it further increased by 24 as shown in Fig. 2d. Histological comparisons of heart sections from LPS-treated (24 h) septic mice with matched controls revealed a moderate infiltration by immune cells following the exposure to LPS (Fig. 2g and Supplementary Fig. 2). However, cardiomyocyte damage was not evident 24 h after exposure to LPS. To monitor the effect of LPS on cardiac function, echocardiography was performed on the day before LPS injection (baseline), and 6 and 24 h after LPS administration (Fig. 2h–j and Supplementary Fig. 3). LPS led to a significant reduction in systolic function as shown by a decreased left ventricular ejection fraction (LVEF) (Fig. 2i) and other parameters (Supplementary Table 2) at 6 h. An increased isovolumetric relaxation time (IVRT) represents prolonged left ventricular relaxation 6 h after LPS administration (Fig. 2j).

### ***LPS induces cardiomyocyte-derived GFP***^+^***mEV release to the circulation***

**Fig. 3:**
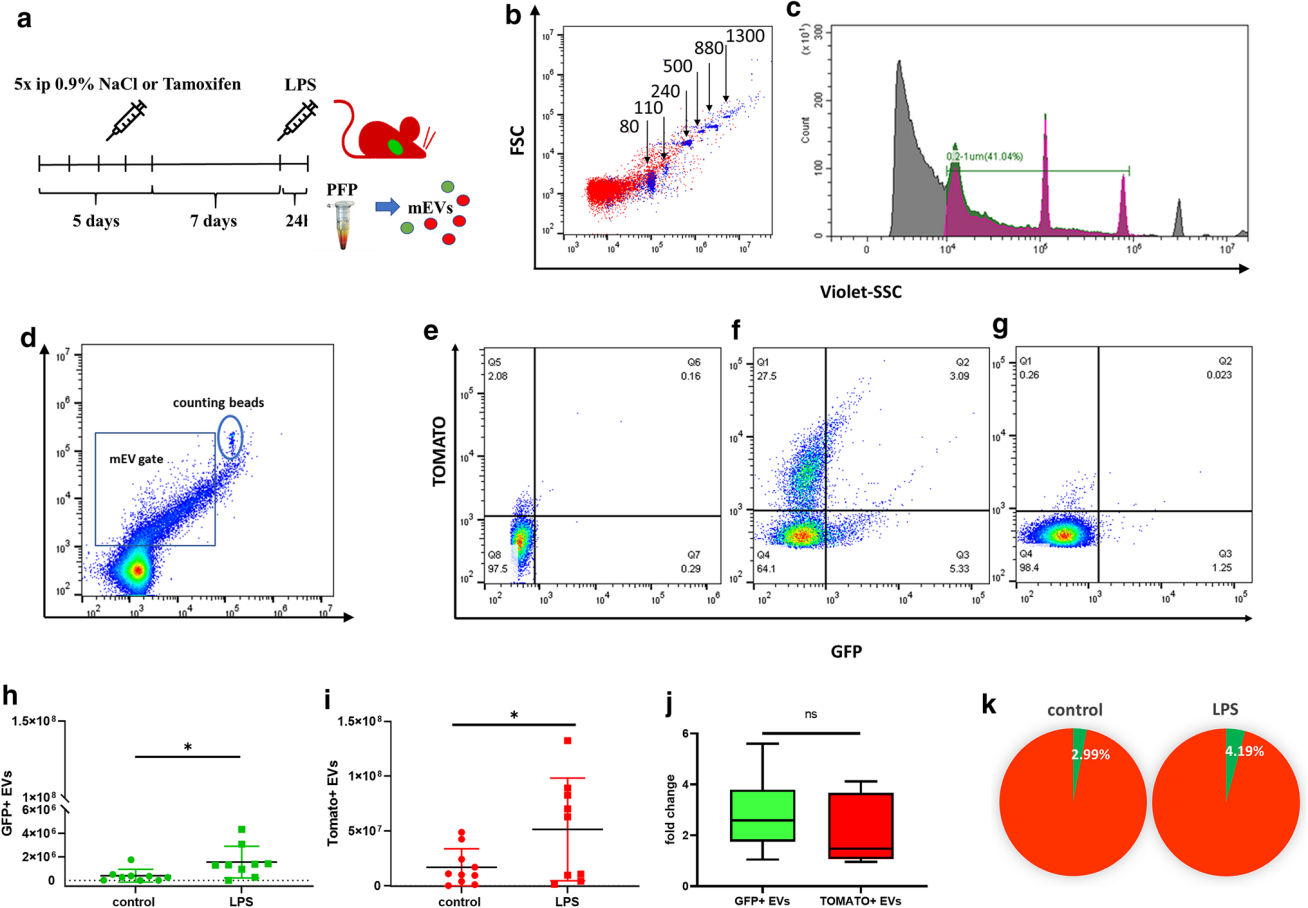
Effect of LPS administration on GFP + EV release to the circulation. **a** Schematic protocol of Tamoxifen induction and LPS injection of mice. **b**, **c** Flow cytometry analysis. Calibration beads were used (0.2–1 µm). **d** The gating strategy for mEVs and the 1 μm counting beads. **e** As a negative control, PFP-derived mEVs were analyzed from C57Bl6 mice. **f** Cardiomyocyte-derived EVs as GFP^+^ events. mEVs secreted by all other cells were Tomato^+^. **g** A 95 ± 1% reduction of the event number after exposure to 0.1% Triton. **h**, **i** Events within the mEV gate were then analyzed, and GFP + events with/without LPS injection are shown on (**h**). **i** demonstrates Tomato^+^ events with/without LPS injection. The figure shows the results of three independent experiments (*n* = 9). The figure represents data from individual mEV samples (mice) as well the means ± SD (*P < 0.05, *T*-test). **j** Fold increase upon LPS treatment, *n* = 6 ns = nonsignificant (*T*-test). **k** The pie diagram represents the percentages of green fluorescent mEVs in control and LPS treated animals (*n* = 9 in each group)

Figure 3a is a schematic illustration of the Tamoxifen induction of Cre gene expression and the LPS injection. Twenty-four hours after the LPS injection, the mice were sacrificed and PFP was prepared. Separated mEVs were analyzed by flow cytometry. For gating, we used PFP-derived mEVs from mTmG mice mixed with calibration beads (0.08–1.3 µm). Initially, we set the gate for the red fluorescent events, and the distribution of the calibration beads in the PE channel is shown in Fig. 3b, c. The substantial reduction of the number of events upon exposure to 0.1% Triton exposure proved the vesicular nature of the separated mEVs. Having shown earlier by fluorescence microscopy that cardiomyocytes from the MerCreMer/mTmG double transgenic mice were GFP positive, we assessed whether GFP positive mEVs were present in the circulation of these mice. As expected, a substantial fraction of the mEVs were Tomato^+^ (Fig. 3d–g). However, we were able to detect a small number of GFP^+^ events (3.9 ± 5.4 × 10^5^/μL) in the control PFP samples. Importantly, LPS increased the release of GFP^+^ mEVs to the circulation (Fig. 3h). We detected 15.6 ± 13.5 × 10^5^/µL plasma (24 h post-treatment; *P* = 0.029; *n* = 9, *T*-test). On the effect of LPS injection, the number of Tomato + mEVs also increased from 1.6 ± 1.6 × 10^7^ (control) to 5.1 ± 4.6 × 10^7^ events/µL plasma (24 h post-treatment; *P* = 0.042; *n* = 9, *T*-test (Fig. 3i). The fold increase of the release of mEVs in mice injected with LPS or with 0.9% NaCl did not differ significantly (Fig. 3j). The pie diagram represents the percentages of GFP^+^ and Tomato^+^ mEVs among plasma mEVs (Fig. 3k). Our attempts to detect GFP-positivity in PFP-derived sEVs bound to latex beads were not successful either in the case of control or in LPS-injected samples.

### *The effect of *in vivo* administered LPS on the release of EVs by adult murine cardiomyocytes*

**Fig. 4:**
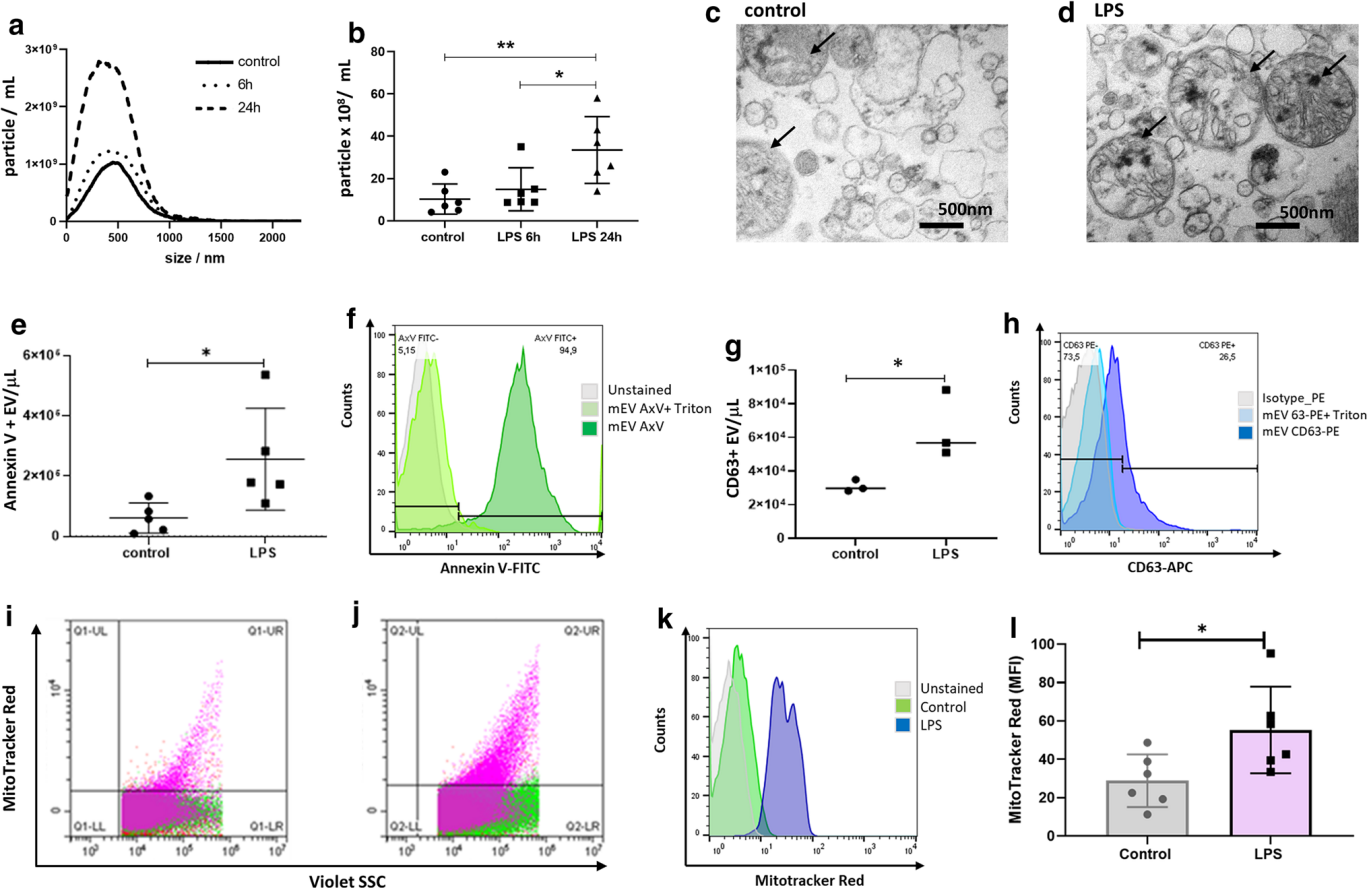
Ex vivo release of mEVs by primary adult murine cardiomyocytes upon in vivo administration of LPS. **a** Representative results of size distribution analysis (NTA) of mEVs from isolated cardiomyocytes from LPS-injected and control mice (24 h conditioned medium). **b** mEVs release was significantly elevated after 24 h. Data are obtained from three independent experiments (mean ± SD values are indicated in the figure) (*P < 0.05; **P < 0.01; *n* = 6, ANOVA). **c**, **d** Transmission electron microscopy of isolated mEVs showing morphological heterogeneity of secreted mEVs and mitochondria (the black arrows point to the mitochondria; scale bar = 500 nm). **e** Annexin V positive events were detected by flow cytometry and the mEV concentrations were determined using counting beads. Data are mean ± SD, *n* = 5, *P < 0.05 vs. control (*T*-test). **f** shows the flow cytometry (FCM) results of Annexin V staining before and after Triton treatment (light green and dark green, respectively); unstained mEVs are in grey. **g** CD63 positive events were detected by flow cytometry and were normalized to EV concentrations using counting beads. Data are mean ± SD, *n* = 3, *P < 0.05 vs. control (*T*-test). **h** FCM results for CD63 before and after Triton treatment (light and darker blue, respectively); grey is for the isotype control. **i**, **j** The mEV fraction was isolated from the conditioned medium of cardiomyocytes from in vivo LPS-induced mice and was stained with MitoTracker Red. The representative dot plot shows the presence of mitochondria in the control **i** and LPS-injected group **j** within the mEV gate. **k** The representative histograms of MitoTracker expression in gated mEV subsets isolated from the control or LPS group are shown green and blue, respectively; unstained mEVs are in grey. **l** Statistical analysis of MitoTracker expression in CMEVs (*n* = 6). Comparisons were performed using a *T*-test, *P < 0.05

Primary cardiomyocyte culture was established from the heart of C57/Bl mice 6 or 24 h after LPS injection. Confirmation of the cardiomyocyte nature of the isolated cells is shown in Supplementary Fig. 4. Cells were cultured in an EV-free serum-containing medium for the next 24 h, and then mEVs were isolated. NTA was used to determine the number of the ex vivo released cardiomyocyte EVs (CMEVs) upon in vivo LPS administration. The size distribution of EVs was also determined (Fig. 4a, b). In cardiomyocyte cultures of LPS-injected mice, the concentration of mEVs was elevated as compared to the controls at 6 h and increased further by 24 h. For further experiments, we selected the 24 h treatment based on the above results.

As shown in Fig. 4c, d, transmission electron microscopy revealed the presence of heterogeneous vesicles and mitochondria in the CMEV preparations in vivo LPS challenge. Molecules of isolated cardiomyocyte-derived EVs were analyzed by flow cytometry, and the presence of mitochondria in the EV preparations was detected by fluorescent MitoTracker Red labeling. Annexin V and anti-CD63 binding was detected on the surface of EVs, and LPS injection-induced the release of both EVs and mitochondria (Fig. 4e–h). Consistent with the findings of Zhang et al. [19], here we also detected specific staining of mitochondria by Mitotracker Red both in the control and the LPS-induced EV fractions indicating the presence of mitochondria in the mEV fractions (Fig. 4i–l).

### Protein cargos of cardiomyocyte-derived mEVs

**Fig. 5:**
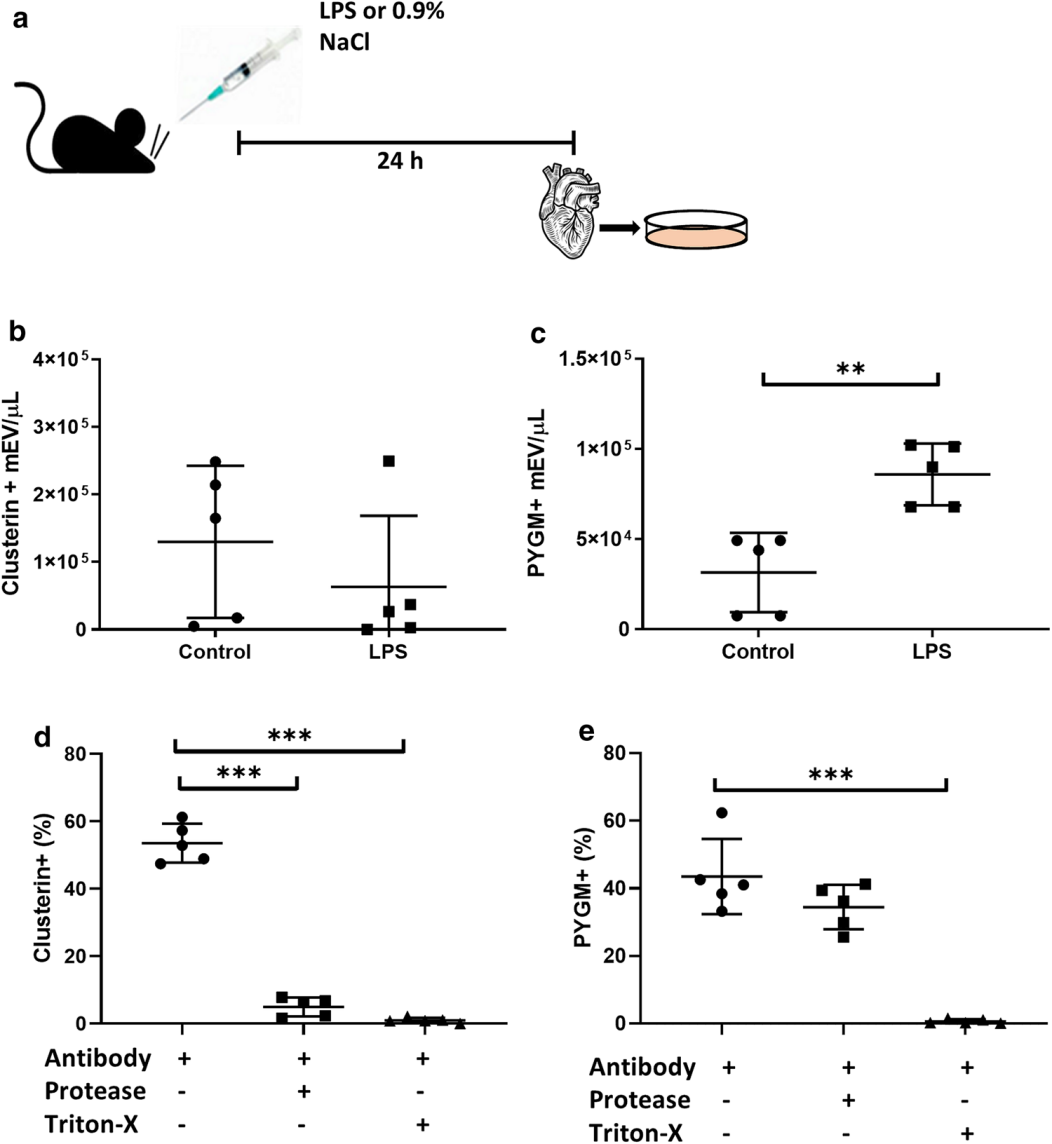
Expression of clusterin and PYGM in CMEVs. **a** Schematic illustration of the experiment. **b**, **c** Clusterin and PYGM levels were measured by flow cytometry. Data are mean ± SD, *n* = 5, **P < 0.01; ***P < 0.001 vs. control. **d**, **e** CMEVs were exposed either to trypsin or Triton X-100 and detected by flow cytometry. Data are mean ± SD, *n* = 6

Based on literature data, we selected two proteins including the non-cardiomyocyte-specific clusterin (ApoJ) [20] and the cardiomyocyte-related muscle glycogen phosphorylase (PYGM) [21], to be tested in CMEVs. To this end, we isolated mEVs from the conditioned media of cultured cardiomyocytes and characterized them by flow cytometry (FACSCalibur, Becton Dickinson). We found that both clusterin and PYGM were present in CMEVs, and the fluorescence intensity of mEVs immunostained for PYGM increased significantly (*P* = 0.0024, *T*-test) upon in vivo LPS injection of mice (Fig. 5).

By now, numerous pieces of evidence suggest that besides transmembrane EV proteins, there are also proteins adsorbed onto the EV surface [22]. Thus, it is important to determine the topology of EV-associated proteins e.g. when considering EVs as biomarkers for cardiovascular disease. To determine if clusterin and PYGM were intravesicular or surface-associated, we exposed CMEVs to trypsin and/or Triton X-100. Trypsin abolished immunostaining for clusterin (a component of the recently identified protein corona formed around EVs [7]) but did not eliminate the signal in the case of the intraluminal protein PYGM (*n* = 5) (Fig. 5d, e).

### Evaluation of the phenotype of mEVs isolated from the PFP of MerCreMer/mTmG mice by flow cytometry

**Fig. 6:**
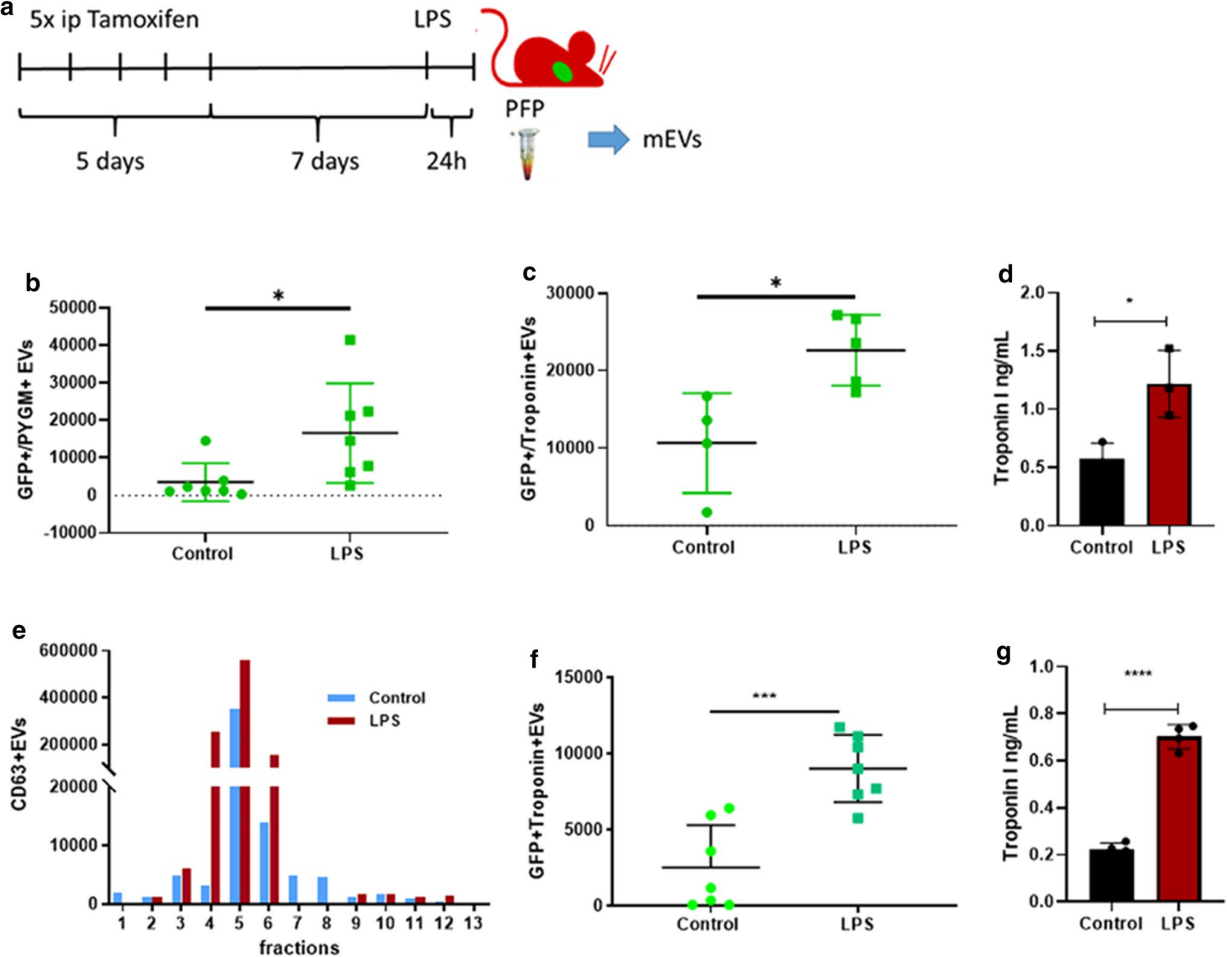
Expression of GFP and cTnI or PYGM on the CMEVs in plasma. **a** Schematic illustration of the experimental setup. **b**, **c** Flow cytometry and ELISA of extracellular vesicles isolated by differential centrifugation from PFP samples. cTnI and PYGM positive events were measured in the GFP gate by flow cytometry. EV concentrations were normalized to counting beads. **d** ELISA shows the results of 24 h LPS injection. Data are mean ± SD, *n* = 3, *P < 0.05 *vs.* control with Student *T*-test. **e** Representative plot showing the SEC elution profile according to CD63 + events/mL as an mEV markers of each SEC fraction. Fractions with the highest CD63 (4–6) were pooled together, to obtain the SEC purified mEV preparations for further investigations. **f**, **g** Flow cytometry and ELISA of extracellular vesicles isolated by SEC from PFP samples. cTnI positive events were measured in the GFP gate by flow cytometry. EV concentrations were normalized to counting beads. **g** ELISA shows the results of 24 h LPS injection. Data are mean ± SD, *n* = 4, ***P < 0.001 vs. control with Student *T*-test

Next, we aimed to detect CMEVs in the circulation (Fig. 6). Therefore, mEV-enriched EV preparations were isolated by differential centrifugation from the PFP of MerCreMer/mTmG mice and were characterized by flow cytometry (Cytoflex). The GFP^+^ CMEVs were positive for PYGM (mean ± SD: 3518 ± 1883 and 16,591 ± 5026 events/µL in the control and the LPS groups, respectively) (Fig. 6b). Importantly, we found in the blood plasma-derived mEV preparation, that GFP + cardiomyocyte mEVs were associated with Troponin-I (cTnI) (mean ± SD: 10,660 ± 3218 and 22,647 ± 2046 events/µL in the control *n* = 4 and the LPS group, respectively) (Fig. 6c). EVs isolated by differential centrifugation were also analyzed with ELISA (Fig. 6d), and we found mean ± SD: 0.57 ± 0.13 ng/mL and 1.21 ± 0.28 ng/mL cTnI in the control and in the LPS groups, respectively (*T* test, *P* = 0.024, *n* = 3). Using SEC for further purification, mEVs from the initial 0.2 mL of platelet-free plasma were finally eluted in 13 fractions, and the CD63 positive fractions were evaluated by flow cytometry (Fig. 6e) Fig. 6f shows that in the pooled fractions 4–6, we found mean ± SD: 2527 ± 1728 GFP + and 9015 ± 2199 cTnI + events/μL in the control and LPS groups, respectively were detected. The SEC purification reduced the average number of vesicles, but the difference between the control and LPS groups became statistically more significant (*T* test, *P* = 0.0004, *n* = 7). The SEC-isolated mEV fraction was also assessed by ELISA (Fig. 6g), and we detected mean ± SD: 0.22 ± 0.02 ng/mL and 0.70 ± 0.05 ng/mL cTnI in the control and in the LPS groups, respectively (*T* test, *P* < 0.0001, *n* = 4).

## Discussion

Inflammation is known to contribute to cardiovascular diseases. To date, the exact pathogenesis of systemic inflammatory syndrome-induced cardiomyopathy is still unclear, and effective treatments for cardiac complications of SIRS are lacking. Thus, animal models are particularly important for investigating SIRS (including sepsis)-associated cardiac damage [23].

In our study, we used transgenic mice expressing a tamoxifen-inducible Cre recombinase protein fused to two mutant estrogen-receptor ligand-binding domains (MerCreMer) under the control of the alpha myosin heavy chain promoter [24]. These mice were crossed with the CAG-tdTomato-flox-membrane GFP-targeted mice to examine the cardiac tissue-specific expression of GFP [25]. We used these mice to study if CMEVs could enter the bloodstream under steady-state and septic conditions. Sepsis is often modeled by intraperitoneal injection of LPS in experimental animals. LPS-induced myocardial dysfunction has been reported in mice [17], and the authors demonstrated that LPS induced elevated serum levels of cardiac Troponin-T (cTnT) and leukocyte infiltration in the myocardium. Here we confirmed that LPS induced systemic inflammation and functional cardiac damage in mice. Upon LPS injection, we found elevated serum levels of tumor necrosis factor-α and altered echocardiographic parameters which indicated inflammation, cardiomyocyte damage, and cardiac dysfunction, respectively.

In line with some previous studies [26–28], here we found that LPS increased the levels of TNF-α, GDF-15, and cTnI in PFP samples of LPS-injected mice. In addition to TNF-α production (which reflects inflammation), GDF-15 and cTnI are indicators of heart damage. cTnI is cardiomyocyte-specific and the presence of cTnI in the circulation is a sensitive biomarker of myocardial damage [29]. Similarly, GDF-15, a pleiotropic cytokine is known as a biomarker of heart failure [30]. It belongs to the TGF-β family [31]. Cardiomyocytes express and secrete GDF-15 after stimulated ischemia and reperfusion in mice, and endogenous GDF-15 protects the heart from ischemic injury [32]. Thus, detection of elevated levels of GDF5 and cTnI confirmed cardiac damage in our LPS model.

In our next approach, we analyzed the conditioned medium of primary murine cardiomyocytes. Unexpectedly, we found a high number of free mitochondria in the conditioned medium of these cells. This finding is, however, not so surprising if we consider that upon mitochondrial stress, cells are known to release mitochondria. This process is considered to be an alternative mitochondrial quality control pathway of cells (distinct from mitophagy) [33]. The mitochondrial release has been also shown for human T-lymphoblastic leukemia, murine fibroblast cells, or activated monocytes [34, 35]. In the conditioned media on cardiomyocytes, in addition to extracellular mitochondria, we identified CMEV of different sizes. EV released by cardiomyocytes has been reported by several groups [19, 36]. It has also been demonstrated that cardiac damage induces an increased EV secretion by cardiomyocytes. When cardiac α-actin–GFP^+^ mice were subjected to myocardial infarction (by coronary artery ligation), there was a transient increase in cardiac tissue-eluted lEVs and sEVs [37]. In line with these previous findings, we detected an increased ex vivo release of cardiomyocyte-derived mEVs 24 h after LPS injection of mice as compared to controls.

Our next and most important question was whether CMEV could cross tissue barriers and enter the systemic circulation. To investigate this question, we analyzed PFP-derived EVs of transgenic mice by flow cytometry. First of all, we could identify a small population of circulating EVs (carrying membrane GFP) in healthy adult mice. These data provide direct evidence that CMEVs could reach systemic circulation even in a steady-state condition. The next obvious question was whether LPS-induced in vivo cardiac damage leads to an increased release of CMEVs to the bloodstream. Our flow cytometry data confirmed that indeed, 24 h after LPS injection, there was a significant increase of green fluorescent events in the mEV gate when analyzing PFP-derived EV preparations. There was also an elevation in the number of TdTomato^+^ mEVs as a sign of systemic inflammation induced by the LPS injection.

Thus, our inducible transgenic mouse model enabled us to identify GFP membrane-positive cardiomyocyte-derived mEVs. We also confirmed that these cardiomyocyte-derived GFP^+^ mEVs harbored cTnI and PYGM. Our findings of the presence of cardiac EVs in the circulation are in line with previous studies showing that that EVs secreted in various solid organs can reach the systemic circulation. Examples include CNS-derived, liver, kidney, lung, and pancreas-derived extracellular vesicles [38–42]. The present study complemented the list of solid organs with the heart from where EVs reach the circulation.

This study was made available using a double transgenic mouse model expressing membrane GFP^+^ cardiomyocytes. Beyond the present study, this model may also help to determine the impact of CMEVs in the cross-talk between cardiomyocytes and other cells both inside and outside of the heart.

The key novel finding of this study that both in a steady-state condition and upon cardiac damage, CMEVs are present in the blood plasma supports the concept that identification of heart-specific circulating EV biomarkers could be a promising and feasible approach to the field of cardiovascular diseases.

## Supplementary Information

Below is the link to the electronic supplementary material.

Supplementary file1 (PDF 767 KB)

## Data Availability

Not applicable.
