# Supplementary material for: Circulating cardiomyocyte-derived extracellular vesicles reflect cardiac injury during systemic inflammatory response syndrome in mice
Source: Cell Mol Life Sci. 2022 Jan 20;79(2):84. doi: 10.1007/s00018-021-04125-w (PMC8776681; doi:10.1007/s00018-021-04125-w)
Supplement: Supplementary file 1 — Supplementary file1 (PDF 767 KB) [file 18_2021_4125_MOESM1_ESM.pdf]

Supplementary Data for

Cellular and Molecular Life Sciences

Circulating cardiomyocyte-derived extracellular vesicles reflect cardiac injury during systemic inflammatory response syndrome in mice

Hargita Hegyesi[1], Éva Pallinger[1], Szabina Mecsei[1], Balázs Hornyák[1], Csenger Kovácsházi[2], Gábor B Brenner[2], Zoltán Giricz[2], Krisztina Pálóczi[1], Ágnes Kittel[3], József Tóvári[4], Lilla Turiak[5], Delaram Khamari[1], Péter Ferdinandy[2,7], Edit I. Buzás[1,6,8]

[1] Semmelweis University, Department of Genetics, Cell- and Immunobiology, Budapest, Hungary

[2] Semmelweis University, Department of Pharmacology and Pharmacotherapy, Budapest, Hungary

[3] Institute of Experimental Medicine, Eötvös Loránd Research Network, Budapest, Hungary

[4] National Institute of Oncology, Department of Experimental Pharmacology, Budapest, Hungary

[5] MS Proteomics Research Group, Research Centre for Natural Sciences, Eötvös Loránd Research Network, Budapest, Hungary

[6] ELKH-SE Immune-Proteogenomics Extracellular Vesicle Research Group, Budapest, Hungary

[7] Pharmahungary Group, H-6722 Szeged, Hungary[8] Hungarian Centre of Excellence for Molecular Medicine (HCEMM) – Semmelweis University Extracellular Vesicle Research Group, Budapest, Hungary

Corresponding author:

Hargita Hegyesi, hegyesi.hargita@med.semmelweis-univ.hu

Supplementary Table I. List of antibodies used for immunofluorescence studies

Antibodies	Conjugate	Supplier	Clone	Species	Dilution
Albumin	No	abcam	ab207327	rabbit	1:100
HSP-70	No	invitrogen	Ma3-006	mouse	1:100
PYGM	FITC	Biorbyt	orb9674	rabbit	1:100
PYGM	No	FineTest	FNab06977	rabbit	1:100
CPN1	No	AntibodyGenie	CAB78887	rabbit	1:100
CLU	No	AntibodyGenie	CAB12913	rabbit	1:100
CD63	APC	Sony	1319525	rat	1:100
CD63	PE	Sony	143903	rat	1:100
CD9	APC	Sony	1224060	rat	1:100
CD81	PE	Sony	1124530	hamster	1:100
CD63	No	Santa Cruze	sc-15363	rabbit	1:50
CD81	No	Sigma	SAB3500454	rabbit	1:50
alpha-actinin	FITC	Frank	106936	human	1:1000
cTnI	biotin	Abbexa	abx272698	rabbit	1:100
Alix	No	Sigma	SAB4200477	rabbit	1:50
Caveolin-3	No	ThermoFisher	PA1-066	rabbit	1:100
Rabbit IgG	APC	abcam	ab232814	rabbit	1:100
Mouse IgG1	FITC	Sony	2630005	mouse	1:500
Armenian hamster	APC	Sony	2604635	hamster	1:100
Rat IgG2a	APC	Sony	2603115	rat	1:100
Rat IgG1	AlexaFluor-647	Sony	2602090	rat	1:100
REA control	FITC	Frank	104611	human	1:100
Rabbit IgG H&L	Cy2	abcam	ab6940	goat	1:1000
Rabbit IgG H&L	APC	ThermoFisher	31984	goat	1:500
Rabbit IgG H&L	AlexaFluor-647	abcam	ab150079	goat	1:500
Mouse IgG	FITC	invitrogen	AB_2536524	goat	1:1000
Mouse IgG	HRPO	invitrogen	AB_2536527	goat	1:10000
Rat IgG	HRPO	invitrogen	AB_228356	goat	1:10000
streptavidin	APC	Sony	2626215		1:1000
AnnexinV	FITC	Sony	3804530		1:500

Supplementary Figure 1 Validation of the EVs based on MISEV proposal

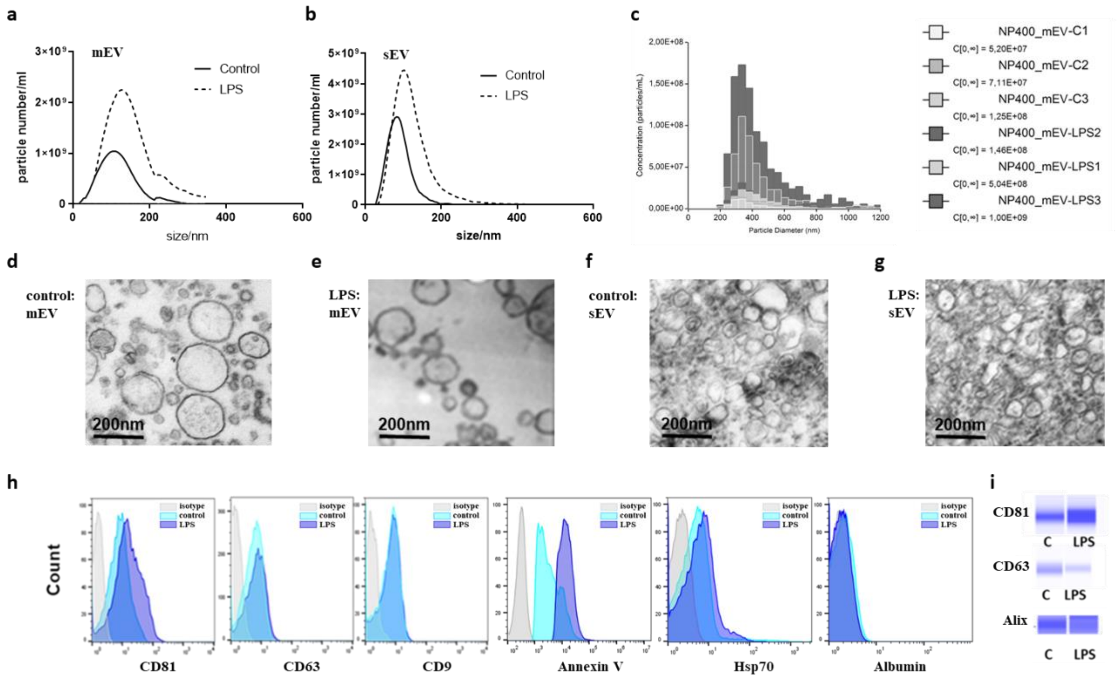

Supplementary Fig. 1 Characterization of the mEVs by the MISEV2018 guidelines [3]
a, b shows concentration and particle sizes of platelet-free plasma EV fractions measured by NTA. **c** Size distibution of a 3-3 samples before and after LPS injection determined by qNano. **d-g** TEM images of control and LPS-challenged samples for both mEV and sEV fractions; **h** Flow cytometry histograms for five positive and one negative EV markers: light grey indicates the isotype control, light blue is the control, and violet shows the LPS group. **i** WES detection of three EV markers CD81= 22kDa, CD63= 30-65kDa, Alix= 95-105kDa.

Supplementary Figure 2. Effect of LPS on inflammatory cell infiltration

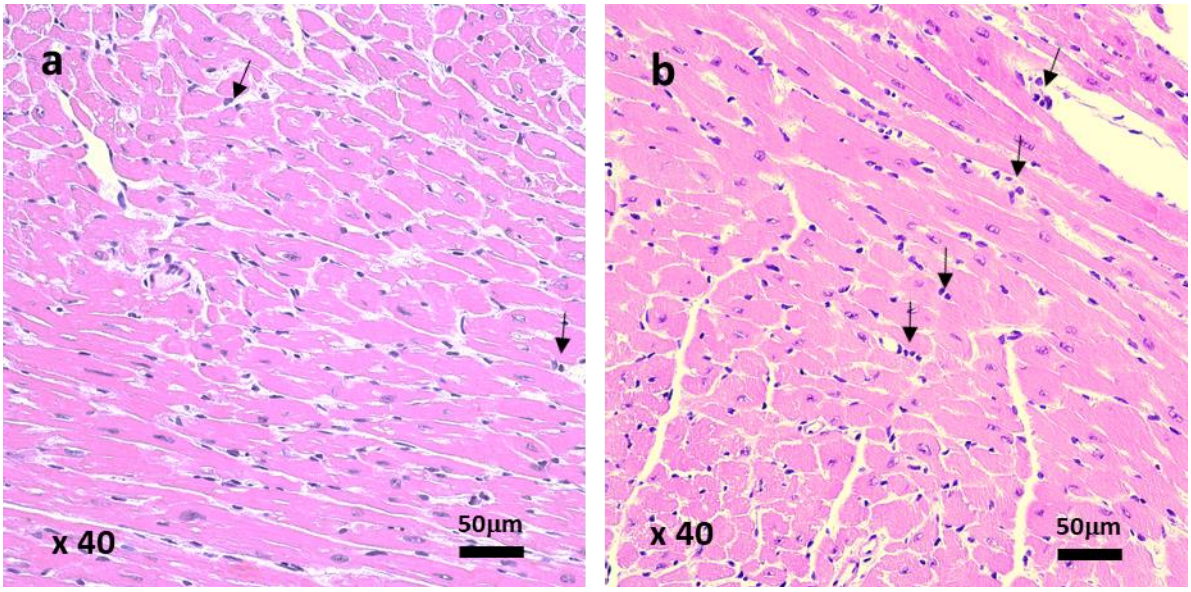

Heart histopathology H&E staining. Panels **a** on the left panel is example of control heart and on the right **b** is the images of the LPS group. Inflammatory cells pointed with black arrows. Magnification 40x (Eclips, Nikon).

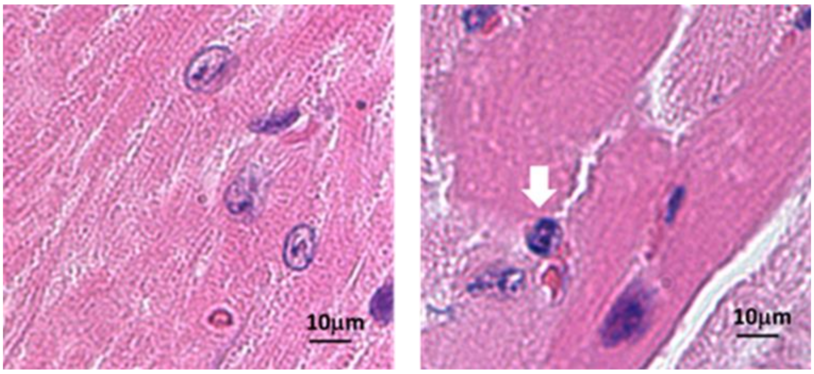

Representative images from formalin-fixed and paraffin-embedded sections (4-µm thickness) of mouse hearts stained with H&E. Twenty four h following LPS injection, diffuse leukocyte infiltration was observed in the hearts. The white arrow points to a granulocyte (objective magnification was 40 X and optical magnification was further 5X).

Supplementary Table 2.

Echocardiographic evaluations were performed one day before (baseline), 6 and 24 hours after intraperitoneal injection of LPS or NaCl (n= 6 mouse/group).

	Baseline				6 hours				24 hours			
	Vehicle		LPS		Vehicle		LPS		Vehicle		LPS	
**BW (g)**	25,34 ± 1,89		23,45 ± 4,01		25,21 ± 1,65		22,64 ± 3,72		25,19 ± 1,76		20,97 ± 3,62	
**HR (b.p.m.)**	529,31 ± 23,43		508,37 ± 188,43		535,08 ± 27,76		516,48 ± 186,74	*#	505,89 ± 47,05		491,05 ± 174,29	
**LV mass (mg)**	97,35 ± 26,25		95,14 ± 31,10		100,70 ± 28,67		107,39 ± 44,99		115,18 ± 22,50		90,91 ± 28,14	
**LVPWd (mm)**	0,84 ± 0,07		0,77 ± 0,28		0,81 ± 0,10		1,12 ± 0,49	*#	0,91 ± 0,07		0,83 ± 0,30	
**LVPWs (mm)**	1,38 ± 0,18		1,21 ± 0,42		1,31 ± 0,14		1,32 ± 0,57		1,35 ± 0,09		1,22 ± 0,43	
**LVAVd (mm)**	1,03 ± 0,15		0,96 ± 0,33		1,03 ± 0,25		0,92 ± 0,27		1,09 ± 0,12		0,95 ± 0,33	
**LVAVs (mm)**	1,72 ± 0,17		1,47 ± 0,50		1,64 ± 0,23		1,20 ± 0,40	*#	1,77 ± 0,20		1,53 ± 0,55	
**LVIDd (mm)**	3,56 ± 0,49		3,74 ± 1,26		3,70 ± 0,47		3,49 ± 1,24		3,74 ± 0,35		3,46 ± 1,21	
**LVIDs (mm)**	1,96 ± 0,57		2,25 ± 0,69		2,19 ± 0,56		2,81 ± 1,03		2,20 ± 0,35		2,03 ± 0,68	
**LVESV (µL)**	16,46 ± 7,71		19,96 ± 7,21		23,40 ± 11,62		32,16 ± 13,90		21,13 ± 11,41		19,34 ± 8,00	
**LVEDV (µL)**	47,69 ± 17,18		51,76 ± 15,48		58,17 ± 16,25		48,01 ± 17,21		55,26 ± 12,95		46,81 ± 15,86	
**FS (%)**	45,81 ± 7,38		39,86 ± 13,13		41,47 ± 7,55		20,41 ± 8,09	*#	41,27 ± 4,53		41,44 ± 14,33	
**SV (µL)**	31,23 ± 9,74		31,80 ± 9,72		34,77 ± 7,21		15,86 ± 5,62	*#	34,13 ± 4,00		27,47 ± 9,35	
**CO (mL/min)**	16,63 ± 5,66		16,08 ± 4,57		18,64 ± 4,21		8,26 ± 3,07	*#	17,22 ± 2,13		13,43 ± 4,49	
**E (mm/s)**	787,77 ± 75,51		818,23 ± 294,19		811,75 ± 65,57		565,91 ± 206,82	*#	751,21 ± 100,20		657,59 ± 217,11	#
**IVCT (ms)**	15,22 ± 2,74		12,41 ± 4,14		12,98 ± 3,25		16,69 ± 5,32		11,97 ± 3,99		12,38 ± 4,68	
**e' (mm/s)**	-26,87 ± 6,12		-27,65 ± 12,98		-26,36 ± 4,60		-18,40 ± 9,68	#	-24,50 ± 5,42		-18,25 ± 9,81	#
**E/e'**	-30,20 ± 5,01		-29,65 ± 13,36		-31,68 ± 6,74		-31,54 ± 15,30		-31,50 ± 5,69		-38,11 ± 19,57	

Two way anova with Tukey post-hoc test. * and # represents $p < 0.05$ significance, * VS Vehicle 6 hours, # VS LPS baseline

Supplementary Figure 3. Representative frames from B-mode imaging (labeled PSLAX)

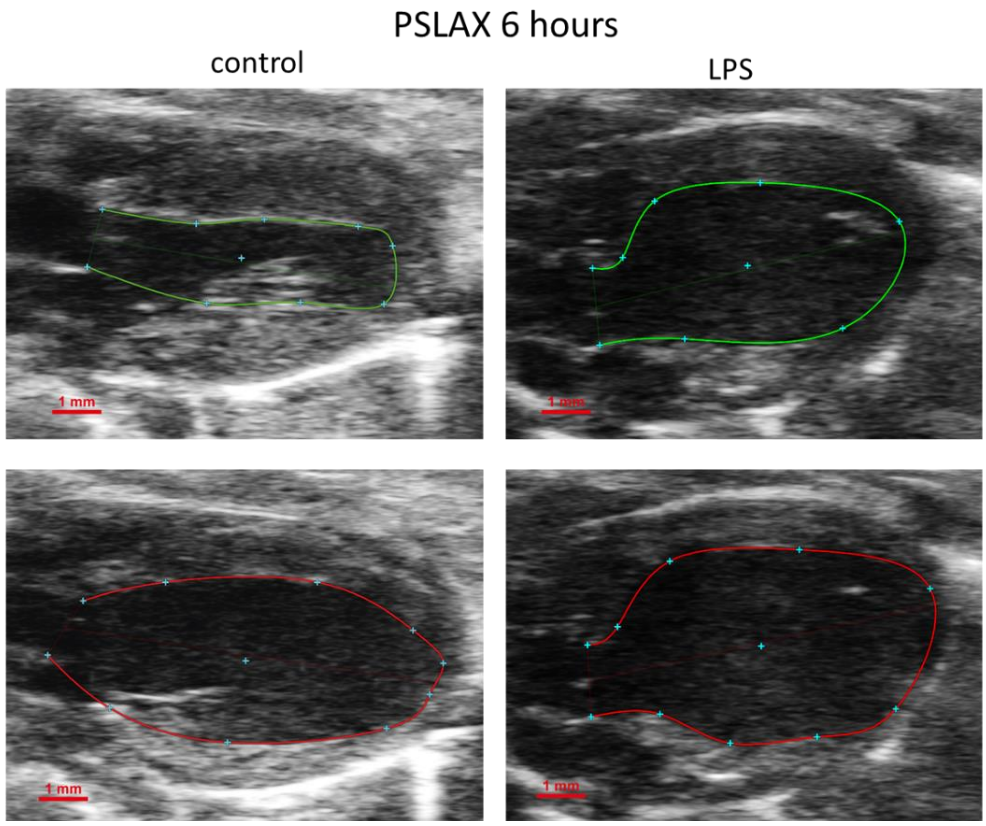

Supplementary Fig. 3 Representative frames from B-mode imaging (labeled PSLAX) are shown for the two (control, LPS) groups.

Supplementary Figure 4. Confirmation of the cardiomyocyte nature of the isolated cells

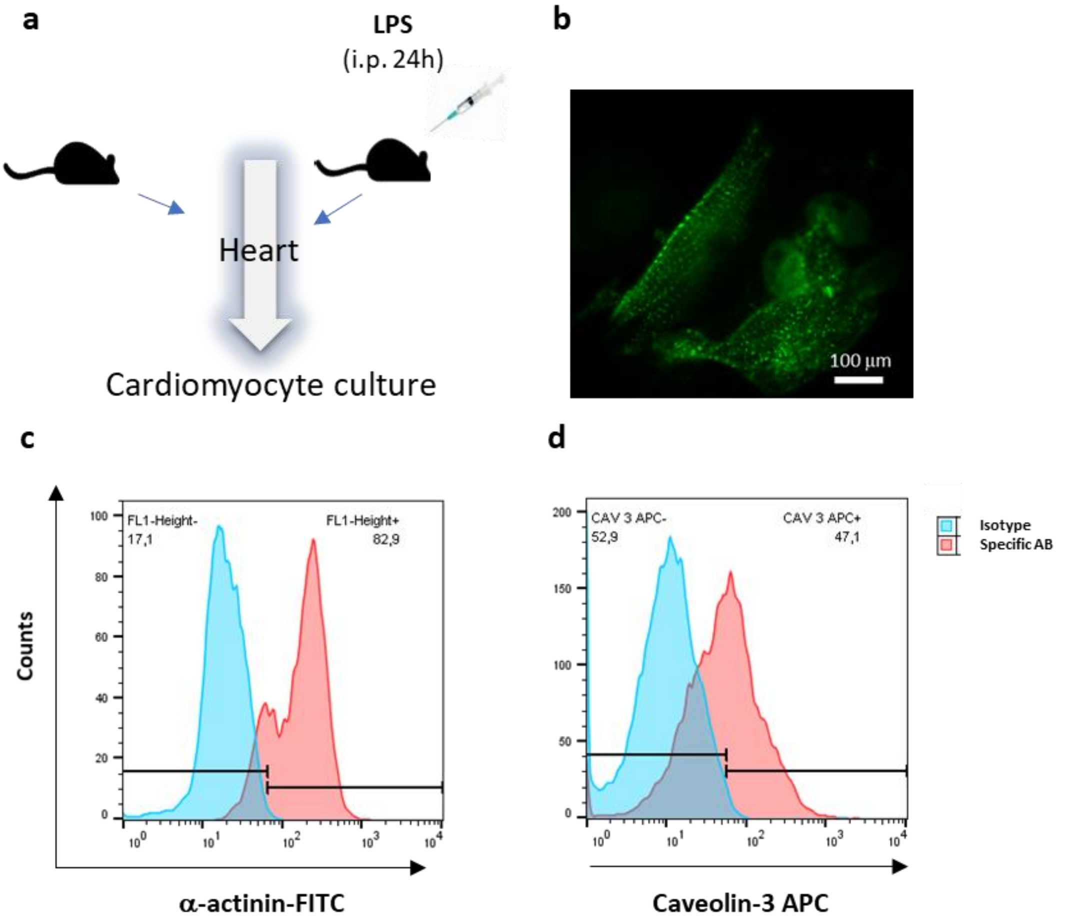

Experimental setup is outlined on Panel (**a**). The microscopy (Eclipse E600, Nikon) image of immunfluorescent stained cultured cardiomyocytes are shown on (**b**); the green fluorescent staining portrays α-actinin-FITC. (**c,d**) representative flow cytometry histograms
